# Dignity and Impudence

**Published:** 1895-01

**Authors:** 


Dignity and Impudence.—We were once much amused at a picture with the above title. It represented a noble, intelligent mastiff, going along with his head and tail up, and with a "far away look in his eye," beset by a miserable little half breed, or no breed, fise, continually snapping at his heels. The fise was evidently beneath the notice of the big dog, and his attentions did not appear to even annoy him.

There has recently occurred in the course of medical journalism in a sister State, an equally striking illustration of the subject. The *Lancet-Clinic*, of Cincinnati, published something, copied from the daily papers of that city, concerning the adulteration of food and the public health. A manufacturing firm saw something in it of more than ordinary interest to them, and put in an order for a large number of reprints of said article, or rather, of extra copies of the *Lancet-Clinic*, for distribution in the interest of their business. In a trial of a druggist which ensued, consequent upon a report of the food commissioners, which report was published in the daily papers, the editor of the *Lancet-Clinic* was put upon the stand, and in answer to questions put to him, related the facts as above: that he had sold to a manufacturing firm 150,000 extra copies of his journal, containing the report of the food commissioners, and some editorial comments on same.

Whereupon a very sore-headed journal of Cincinnati,—the one which championed and defended the Amick layout (that is sufficient to give an idea of the ethical status of said journal),—devotes nearly an entire issue to attempts at ridiculing Dr. Culbertson, and calls the sale of extra copies of his journal the "sale of a soul."

That is pretty far fetched. It is astonishing to what depths certain disappointed, unsuccessful and envious rivals will descend in their frantic endeavors to besmirch their betters..

The sale of extra copies of a journal, or of reprints, in any number, needs no defense. It is perfectly legitimate; Dr. Culbertson had as much right to sell 150,000 copies as he had to sell one, and the sale of such a quantity indicates that it was *worth buying*, or the parties would not have bought it; it may be considered a pretty shrewd business transaction.

The sore-headed journal is simply jealous. Nothing has ever appeared in its columns to make anybody want more than a single copy,—and one copy filled with such spleen and ill-nature as the one referred to is more than enough for anybody. As the editor is careful enough in his language to keep safely within bounds of the libel law, Culbertson just goes straight along, not noticing the little rival, snapping at him, any more than did the *other* big dog referred to above notice the little fise; not even condescending to notice him.

The only thing we see in the transaction to condemn is, Dr. Culbertson should have known better than to mail this extra edition as second class matter; he should have known—and if he did not, his ignorance is inexcusable—that they should have been uniform with his regular edition to be entitled to the rates. It seems to us if we had filled an order for reprints or for extra copies, we should have delivered them by express to the purchasers; mailing them, unless it was a part of the contract, should have been their business.

But they couldn't make Culbertson tell what he realized from his sale,—that's what's the matter with the little sore-head; that might be called the soul of the sale, which is about as near as any unprejudiced person can come, upon reading the facts, to seeing any "sale of soul," or anything else unbecoming a legitimate journal.

The *Lancet-Clinic* is a success; it cannot afford to notice everything that barks at it.

